# Reclassification of SGA and EUGR in very preterm infants after the introduction of Fenton-2025: a retrospective cohort comparison with INTERGROWTH-21st and Fenton-2013

**DOI:** 10.1186/s12887-026-07215-5

**Published:** 2026-06-25

**Authors:** Kutlay Gür, Sevilay Topcuoğlu, Emre Dincer, Elif Özalkaya, Güner Karatekin

**Affiliations:** 1 Department of Pediatrics, Zeynep Kamil Women and Children’s Diseases Training and Research Hospital, University of Health Sciences Türkiye, Istanbul, 34668 Türkiye; 2 Division of Neonatology, Department of Pediatrics, Zeynep Kamil Women and Children’s Diseases Training and Research Hospital, University of Health Sciences Türkiye, Istanbul, Türkiye

**Keywords:** Extrauterine Growth Restriction, Infant, Very Premature, Growth Charts, Fenton Growth Chart, INTERGROWTH-21st, Small for Gestational Age

## Abstract

**Background:**

In very preterm infants, the classification of small for gestational age (SGA) and extrauterine growth restriction (EUGR) depends on the growth chart used. In this context, the classification implications of the newly introduced Fenton-2025 curves have not yet been evaluated in this population. We compared SGA and EUGR classification across Fenton-2025, INTERGROWTH-21st, and Fenton-2013, and examined chart-specific associations between EUGR and major neonatal morbidities.

**Methods:**

This retrospective single-centre cohort study included 272 infants born at 24–32 weeks’ gestation. SGA was defined as birth weight below the 10th percentile. EUGR was defined as discharge weight-for-age Z score below − 2. Associations between chart-defined EUGR and neonatal morbidities were examined using multivariable logistic regression adjusted for gestational age, birth weight, and sex.

**Results:**

SGA rates differed in rank order across the three charts, being lowest with Fenton-2013 (9.9%), intermediate with INTERGROWTH-21st (15.8%), and highest with Fenton-2025 (24.3%). For EUGR, the ordering was different: rates were lowest with INTERGROWTH-21st (26.1%), intermediate with Fenton-2013 (33.5%), and highest with Fenton-2025 (40.8%). Despite this difference in rank order, the three charts showed a nested classification pattern. Compared with INTERGROWTH-21st, Fenton-2025 classified 56% more infants (n = 40) as EUGR. Nevertheless, in adjusted analyses, infants classified as EUGR according to the Fenton 2013 and INTERGROWTH-21st charts were associated with higher adjusted odds (aOR 6.37, 95% CI 2.07–19.57, and aOR 5.03, 95% CI 1.82–13.90, respectively) of necrotising enterocolitis (≥ stage II) compared to non-EUGR infants, and this higher adjusted odds persisted after reclassification using the Fenton 2025 chart (aOR 4.96, 95% CI 1.68–14.64).

**Conclusions:**

Fenton-2025 applies a more inclusionary approach in classifying very preterm infants as SGA and EUGR compared with INTERGROWTH-21st and Fenton-2013. The clinical implications of this broader classification require further outcome-linked validation.

**Supplementary Information:**

The online version contains supplementary material available at https://doi.org/10.1186/s12887-026-07215-5.

## Background

Accurate and individualised monitoring of growth in preterm infants is fundamental to optimal care because early growth is associated with long-term neurodevelopmental, metabolic, and physical health [1]. Growth charts are the main tools used for this follow-up and combine gestational age, sex, and anthropometric data; to enable consistent, standardised assessment of preterm growth and comparability across populations, international charts have been developed, including the Fenton-2013, INTERGROWTH-21st (IG-21), and Fenton-2025 growth charts [2–4]. Despite their widespread use, no consensus exists on which chart best assesses growth in preterm infants [2, 5, 6].

The Fenton-2013 curves were derived from nearly four million infants born at 22–50 weeks’ gestation and represent a fetal-based reference. However, their limited demographic diversity and their inability to reflect early postnatal physiological fluid shifts have raised concerns regarding their suitability for assessing extrauterine growth [7]. In contrast, the IG-21 project introduced an international prescriptive standard based on prospectively collected data from healthy preterm infants up to 64 weeks’ postmenstrual age [3]. Although IG-21 offers a physiologically grounded postnatal trajectory, several studies have suggested that its sensitivity for detecting small-for-gestational-age (SGA) infants may be limited in some populations [8, 9], potentially leaving some infants at increased perinatal risk unlabelled in term populations [10].

Across studies, the two growth charts differ most notably in two diagnostic areas: (i) SGA classification and (ii) extrauterine growth restriction (EUGR). For SGA, IG-21 tends to classify a higher proportion of infants as SGA, especially in very preterm cohorts [5, 11, 12]; whereas Fenton-2013 charts report higher SGA rates in late preterm and term populations [13, 14]. For EUGR, Fenton-2013 typically yields higher rates [5, 6, 11], whereas IG-21 often identifies fewer cases that may be more strongly associated with morbidity [5, 6].

The recent introduction of the Fenton-2025 curves represents an effort to address these discrepancies [4]. By excluding pregnancies complicated by maternal hypertension, diabetes, fetal pathology, or provider-initiated early delivery, the updated curves are based on a cleaner dataset of 4.8 million births, including 174,184 infants born before 30 weeks’ gestation. The upward revision of lower percentiles and closer alignment with WHO standards suggest that both SGA and EUGR prevalence may increase when the new chart is used [4].

To our knowledge, comparisons of Fenton-2025 with IG-21 and Fenton-2013 in very preterm infants remain limited. This study addresses this gap by examining how these three charts classify SGA and EUGR in infants born at 24–32 weeks’ gestation and by evaluating the clinical characteristics and morbidity profiles of reclassified infants.

## Methods

### Study design and population

**Fig. 1:**
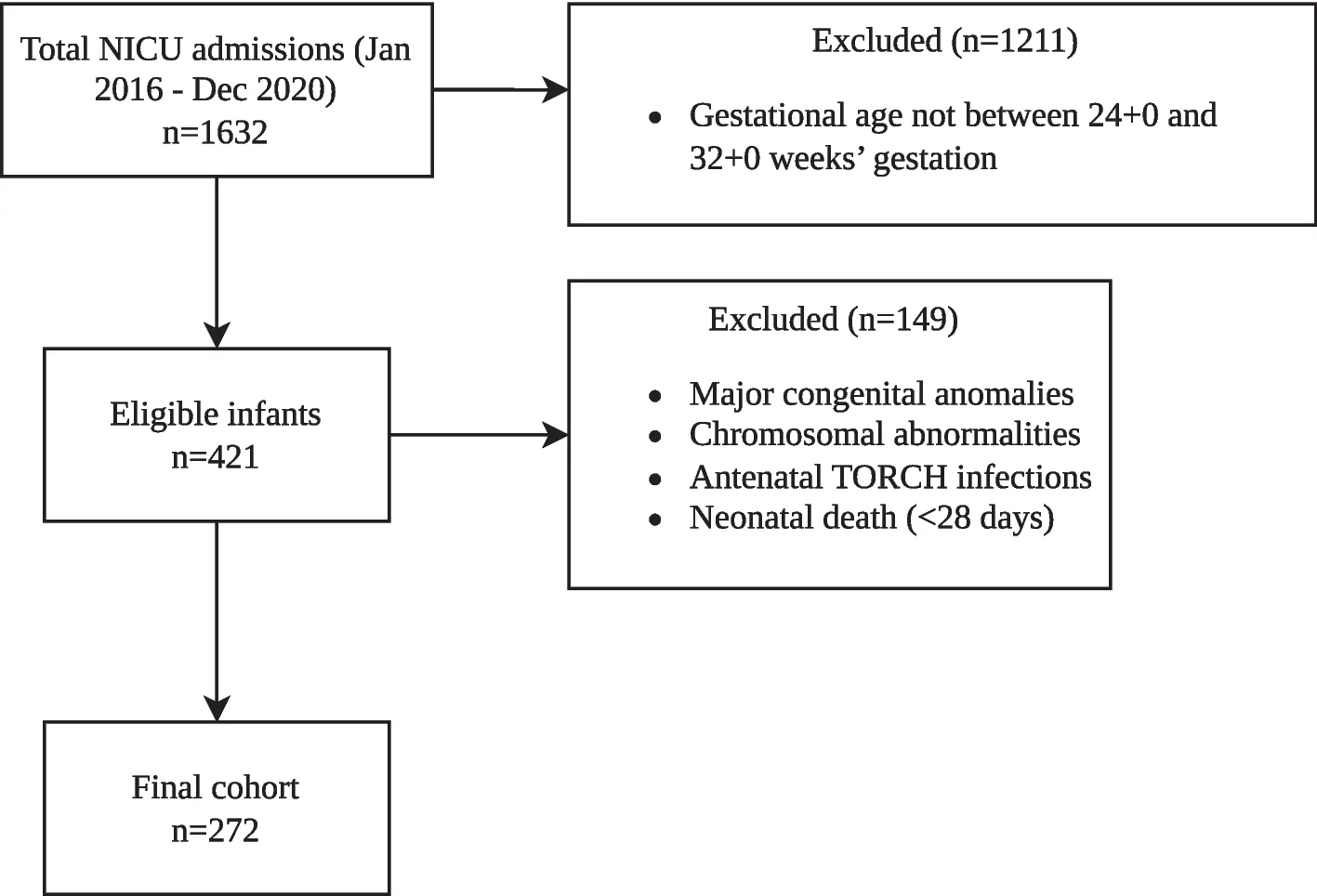
Patient flow diagram of the preterm cohort included in the study. *Abbreviations:* NICU, neonatal intensive care unit; TORCH, toxoplasmosis, other agents, rubella, cytomegalovirus, and herpes simplex virus

This retrospective cohort study was conducted in a tertiary-level neonatal intensive care unit (NICU) at Zeynep Kamil Women and Children’s Diseases Training and Research Hospital, Istanbul, Türkiye, between January 2016 and December 2020. All infants born at 24 + 0 to 32 + 0 weeks’ gestation and admitted during the study period were eligible for inclusion. The exclusion criteria were chromosomal abnormalities, major congenital malformations, antenatal TORCH infection, and death within the first 28 postnatal days. The final analytic cohort comprised 272 infants (Fig. 1).

Gestational age was obtained from obstetric records. Dating was based on the last menstrual period and was corroborated by first- or second-trimester ultrasonography when available. In case of discrepancy, the best obstetric estimate recorded in the chart was used. Clinical and anthropometric data were collected retrospectively from birth/admission until discharge. Retrospective data collection was approved by the Clinical Research Ethics Committee of Zeynep Kamil Women and Children’s Diseases Training and Research Hospital on 21 April 2021 (Decision No. 93), and the updated Fenton-2025 reanalysis was approved by the Zeynep Kamil Hospital Non-Interventional Research Ethics Committee, on 25 February 2026 (Decision No. 26). The study was conducted in accordance with the Declaration of Helsinki.

### Anthropometric assessment and growth classification

Birth anthropometric measurements were extracted from standardised nursing and physician documentation. Birth weight was measured using calibrated electronic scales according to unit protocol and recorded in the medical records shortly after birth. Discharge weight was recorded on the day of discharge. Z scores and percentiles were calculated using PediTools for the Fenton 2013 and Fenton 2025 growth references and the IG-21 bulk calculator for IG-21 standards [15, 16].

Birth weight classification was defined as small for gestational age (SGA; < 10th percentile), appropriate for gestational age (AGA; 10th–90th percentile), and large for gestational age (LGA; > 90th percentile). Extrauterine growth restriction (EUGR) was defined as a weight-for-age Z-score of < –2 at discharge. A cross-sectional definition was used because delta Z-score definitions are less comparable across charts with different baseline distributions.

### Perinatal and neonatal variables

Perinatal risk factors abstracted from obstetric records included preeclampsia, gestational diabetes, antenatal fetal growth restriction (IUGR), abnormal umbilical artery Doppler (absent/reversed end-diastolic flow), chorioamnionitis, preterm premature rupture of membranes (PPROM), and oligo-/polyhydramnios. Neonatal variables included sex, multiple births (singleton vs. multiple), length of NICU stay, duration of invasive and noninvasive ventilation, duration of supplemental oxygen therapy, total parenteral nutrition** (**TPN) duration, day of enteral feeding initiation, day of achieving full enteral feeding, maximum weight loss (%), day of nadir weight, and days to regain birth weight.

### Neonatal morbidities

Neonatal morbidities were defined a priori using standard diagnostic criteria. Sepsis was defined as at least one episode of clinically diagnosed sepsis documented by the treating team during hospitalisation. Retinopathy of prematurity (ROP) was classified according to the International Classification of Retinopathy of Prematurity [17] and analysed as ≥ stage II. Intraventricular haemorrhage (IVH) was graded using the Papile/Volpe classification [18] and analysed as ≥ grade II. Patent ductus arteriosus (PDA) was recorded echocardiographically as a binary variable because severity and treatment details were not consistently available. Necrotising enterocolitis (NEC) was staged according to the modified Bell criteria and analysed as ≥ stage II [19]. Bronchopulmonary dysplasia (BPD) was defined as oxygen requirement on postnatal day 28 and ongoing oxygen or respiratory support at 36 weeks’ postmenstrual age [20].

### Statistical analysis

A minimum sample size of 240 infants was determined a priori to detect 10-percentage-point differences in SGA and EUGR classification rates across growth charts, with 80% power and a two-sided α of 0.05. Statistical analyses were performed using SPSS version 26.0 (IBM Corp., Armonk, NY, USA). Continuous variables are reported as medians (interquartile range), and categorical variables are reported as counts and percentages. Group comparisons used the Mann–Whitney U test or chi-square test, with Fisher’s exact test when expected cell counts were < 5. Differences in classification rates across the three growth charts were evaluated using Cochran’s Q test for related samples, followed by post-hoc pairwise McNemar tests with Bonferroni correction when significant; the corrected significance threshold was p < 0.017. Pairwise agreement between the growth charts was quantified using observed agreement (Po), Cohen’s kappa (κ) with 95% confidence intervals, and prevalence-adjusted bias-adjusted kappa (PABAK). κ values were interpreted according to the Landis and Koch benchmarks [21]. Adjusted associations between EUGR (defined separately by each growth chart) and neonatal morbidities were examined using separate binary logistic regression models for each morbidity, including gestational age, birth weight, and sex as covariates. Adjusted odds ratios (aOR) with 95% confidence intervals were reported. Analyses were conducted using complete-case data for each model. Two-sided p-values < 0.05 were considered statistically significant. A singleton-only sensitivity analysis was performed to address potential non-independence among siblings from multiple pregnancies.

## Results

The study included 272 preterm infants with a median gestational age at birth of 29 + 0/7 weeks (IQR: 27^+0/7^–30^+6/7^) and a median birth weight of 1140 g (IQR: 848–1448). The median maternal age was 30 years (min–max:17–49). The median postmenstrual age at discharge was 36 + 6/7 weeks (IQR: 35^+4/7^–39^+0/7^). The baseline antenatal, perinatal, and neonatal characteristics, as well as major morbidities, are summarised in Table 1.

**Table 1:** Antenatal, perinatal, clinical characteristics and morbidities of the study cohort

Antenatal and Obstetric Characteristics	n(%)
Preeclampsia	59/267 (22.1)
PPROM	59/267 (22.1)
IUGR^a^	40/267 (15.0)
Abnormal Umbilical Cord Blood Flow	35/267 (13.1)
Chorioamnionitis	23/267 (8.6)
Oligo-/Polyhydramnios	29/267 (10.9)
Gestational Diabetes Mellitus	7/267 (2.6)
Pregestational Diabetes Mellitus	5/267 (1.9)
Antenatal corticosteroid administration	186/272 (68.4)
Complete antenatal corticosteroid course^b^	141/272 (51.8)
Perinatal Characteristics	
Cesarean Delivery	205/272 (75.4)
Male Sex	133/272 (48.9)
Multiple birth (twins, triplets)	64/272 (23.5)
Neonatal Morbidities	
RDS	184/272 (67.6)
Early-Onset Sepsis	127/272 (46.7)
Late-Onset Sepsis	161/272 (59.2)
BPD	108/272 (39.7)
PDA	89/272 (32.7)
ROP^c^	45/272 (16.5)
NEC^d^	29/272 (10.7)
IVH^e^	18/272 (6.6)
Clinical Course	
Received Invasive MV	186/272 (68.4)
Received Non-invasive MV	262/272 (96.3)
Received TPN	250/272 (91.9)
Postnatal systemic dexamethasone therapy	49/272 (18.0)
	**Median (IQR)**
Hospital Stay, days	56 (33.8–80.5)
Day of Enteral Feeding Initiation	2 (1–3)
TPN Duration, days	17 (10–26)
Invasive MV Duration, days	8 (3–30.5)
Non-invasive MV Duration, days	6 (3–14)

^a^IUGR, antenatal intrauterine growth restriction, as documented in obstetric records, ^b^ ≥ 2 documented doses administered, ^c^ROP ≥ stage 2, ^d^NEC ≥ stage 2, ^e^IVH ≥ grade 2

*Abbreviations:* *BPD* bronchopulmonary dysplasia, *IVH* intraventricular hemorrhage, *IUGR* intrauterine growth restriction, *MV* mechanical ventilation, *NEC* necrotising enterocolitis, *PDA* patent ductus arteriosus, *PPROM* preterm premature rupture of membranes, *RDS* respiratory distress syndrome, *ROP* retinopathy of prematurity, *TPN* total parenteral nutrition

Based on birthweight, the proportions of SGA, AGA, and LGA infants were 9.9%, 82.7%, and 7.4% according to the Fenton-2013 chart; 15.8%, 76.1%, and 8.1% according to the IG-21; and 24.3%, 66.1%, and 9.6% according to Fenton-2025, respectively. For SGA classification, Cochran’s Q test demonstrated a significant overall difference among the three growth charts (Q = 59.1, df = 2, p < 0.001).

**Fig. 2:**
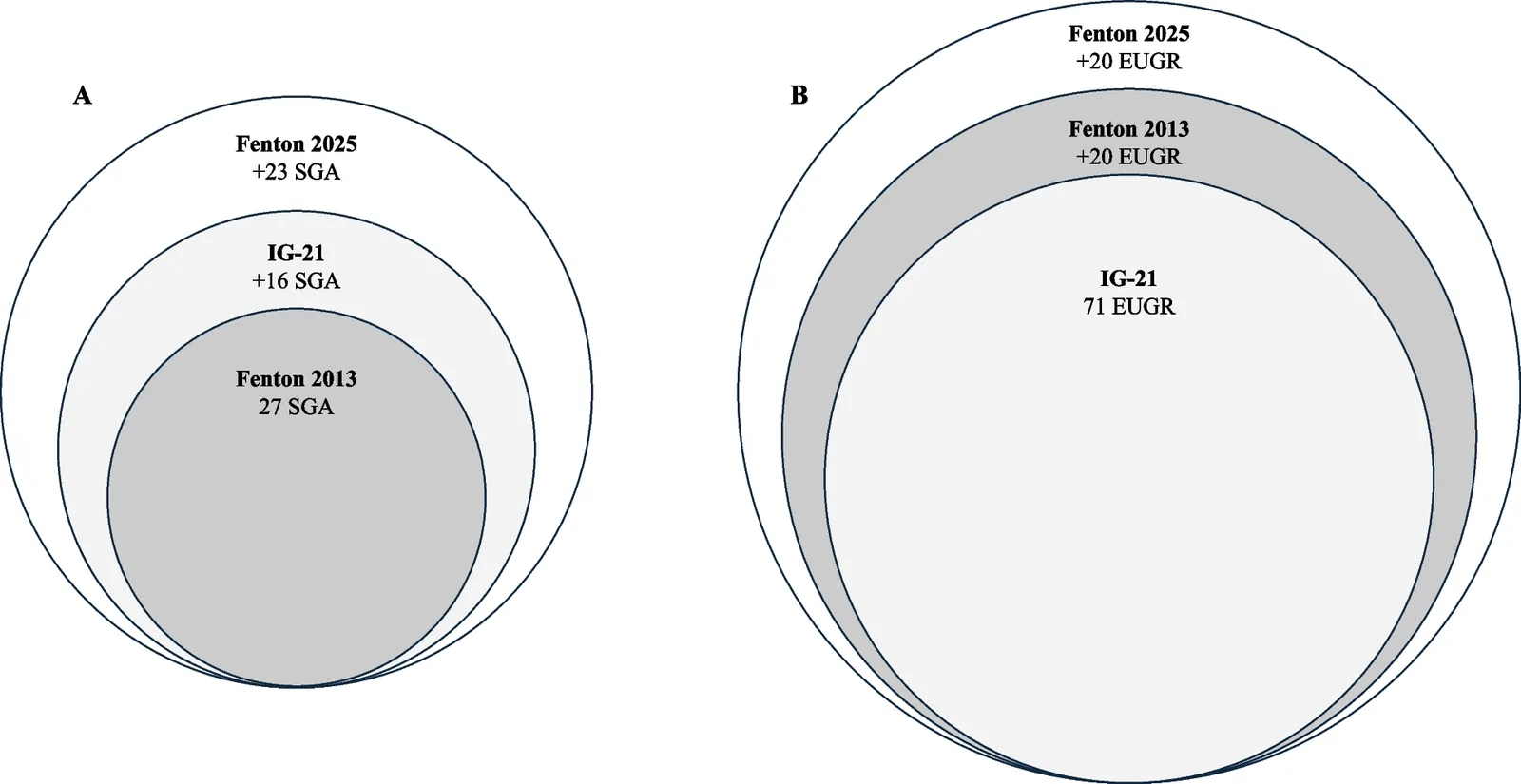
Nested reclassification of SGA at birth and EUGR at discharge across growth charts. The three growth charts yielded different diagnostic rates for both SGA and EUGR, forming a nested reclassification structure in which higher-yield charts encompassed all cases identified by lower-yield charts and added further cases. **A** Fenton-2013 identified 27 infants as SGA; IG-21 added 16 further cases, and Fenton-2025 added a further 23. **B** IG-21 identified 71 infants with EUGR; Fenton-2013 captured these and added 20 more, while Fenton-2025 added 20 additional cases beyond Fenton-2013. *Abbreviations:* F13, Fenton-2013; IG-21, INTERGROWTH-21st; F25, Fenton-2025

At discharge, the median weight-for-age Z scores were − 1.16 (IQR: − 2.09 to − 0.30) for IG-21, − 1.56 (IQR: − 2.35 to − 0.86) for Fenton-2013, and − 1.76 (IQR: − 2.71 to − 0.99) for Fenton-2025. The discharge Z-score distributions differed significantly across the three growth charts (Friedman test, p < 0.001), and all pairwise differences remained significant after Bonferroni correction (all p < 0.001). Both Fenton-2013 (33.5%) and Fenton-2025 (40.8%) identified a greater proportion of infants as EUGR compared with IG-21 (26.1%). All 71 infants classified as EUGR by IG-21 were also classified as EUGR by both Fenton charts. However, Fenton-2013 classified an additional 20 infants as EUGR, resulting in a total of 91 cases. Fenton-2025 further increased this number by identifying 20 additional infants beyond those classified by Fenton-2013, yielding a total of 111 EUGR cases in the study. Consequently, 36% of infants classified as EUGR according to Fenton-2025 were considered normal by IG-21 classification. For EUGR classification at discharge, Cochran’s Q test demonstrated a significant overall difference among the three growth charts (Q = 60.0, df = 2, p < 0.001). Post-hoc pairwise McNemar tests showed that all pairwise differences remained significant after Bonferroni correction for both SGA and EUGR classifications (all p < 0.001). The nested overlap structures for SGA at birth and EUGR at discharge are illustrated in Fig. 2A and Fig. 2B, respectively.

Growth chart definitions showed high pairwise agreement for both size at birth (SGA/AGA/LGA) and EUGR classifications. For SGA/AGA/LGA, agreement was substantial (κ = 0.62–0.76; Po = 0.835–0.915), and PABAK values ranged from 0.753 to 0.873. For EUGR classification, the agreement was substantial to almost perfect (κ = 0.68–0.84; Po = 0.853–0.926) with PABAK values ranging from 0.706 to 0.853. The full agreement metrics and bootstrap-derived 95% confidence intervals are provided in Additional file 1.

Across all three growth charts, no statistically significant differences were observed between SGA and non-SGA infants in neonatal morbidities (all p > 0.05), except for early- or late-onset sepsis (≥ 1 episode), which was consistently more frequent among SGA infants: 100.0% (27/27) with Fenton-2013, 97.7% (42/43) with IG-21, and 95.5% (63/66) with Fenton-2025, whereas in non-SGA infants, it ranged from 81.5% to 83.2% (p = 0.019, 0.009, and 0.005, respectively).

Across all chart definitions, EUGR infants had a more intensive clinical course and a higher burden of major morbidities than non-EUGR infants (Table 2). Across all chart definitions, EUGR infants had longer IMV and NIMV durations, longer TPN duration, and longer hospitalisation. Infants classified as EUGR also showed higher rates of sepsis, RDS, and NEC. BPD and oxygen support were significantly higher in EUGR infants defined by the Fenton-2013 and IG-21, but not by the Fenton-2025. PDA, ROP, and IVH were consistently more frequent in EUGR infants but did not reach statistical significance, except for IVH in the Fenton-2013 defined group.

**Table 2:** Comparison of clinical characteristics and morbidities between EUGR and non-EUGR infants across growth charts

Variable	non-EUGR (n = 161)	F13 EUGR (n = 91)	IG-21 EUGR (n = 71)	F25 EUGR (n = 111)
Duration (days)	Median (IQR)	Median (IQR)	Median (IQR)	Median (IQR)
IMV	7 (3–25)	15.5 (3–48.5)*	16.5 (3–54.3)*	12 (3–41)*
NIMV	6 (2–12.3)	9 (4–19.5)*	10 (4–19.3)*	8 (3.3–17)*
O2 support	5 (1–15)	8 (3.8–19)*	8 (3.8–18.3)*	7 (3–18)
TPN	13 (8–21)	24.5 (17–38.3)*	26 (18–39)*	22 (15–33)*
Hospitalization	46 (29–73.5)	74 (56–100)*	74 (59–104)*	66 (49–93)*
Morbidity	n (%)	n (%)	n (%)	n (%)
Sepsis	129 (80.1)	86 (94.5)*	67 (94.4)*	102 (91.9)*
RDS	98 (60.9)	74 (81.3)*	60 (84.5)*	86 (77.5)*
BPD	56 (34.8)	50 (54.9)*	39 (54.9) *	52 (46.8)
PDA	51 (31.7)	34 (37.4)	24 (33.8)	38 (34.2)
ROP^a^	22 (13.7)	19 (20.9)	17 (23.9)	23 (20.7)
NEC^b^	7 (4.3)	21 (23.1)*	18 (25.4)*	22 (19.8)*
IVH^c^	8 (5.0)	10 (11.0)*	7 (9.9)	10 (9.0)

^a^ROP ≥ stage 2, ^b^NEC ≥ stage 2, ^c^IVH ≥ grade 2, * p < 0.05, compared to the corresponding chart-specific non-EUGR group

Note: Fenton-2025 non-EUGR group is displayed for descriptive reference. For each EUGR column, statistical comparisons were performed against the corresponding chart-specific non-EUGR group

*Abbreviations:* BPD, bronchopulmonary dysplasia; EUGR, extrauterine growth restriction; IG-21, INTERGROWTH-21st; IMV, invasive mechanical ventilation; IVH, intraventricular hemorrhage; NEC, necrotising enterocolitis; NIMV, non-invasive mechanical ventilation; PDA, patent ductus arteriosus; RDS, respiratory distress syndrome; ROP, retinopathy of prematurity; TPN, total parenteral nutrition

In additional comparisons, antenatal corticosteroid administration and postnatal systemic dexamethasone therapy did not differ significantly between EUGR and the corresponding chart-specific non-EUGR groups across the three growth-chart definitions: antenatal corticosteroid administration was 70.4%–72.5% in EUGR infants and 66.3%–67.7% in non-EUGR infants; the corresponding rates for postnatal systemic dexamethasone therapy were 18.9%–23.9% and 15.9%–17.4%, respectively (all p > 0.05).

In the adjusted logistic regression models, including gestational age at birth, birth weight, and sex (Table 3), EUGR remained associated with NEC ≥ stage 2 across all growth-chart definitions (aORs 4.96–6.37), with similar effect sizes across charts, although the point estimate was highest for the Fenton-2013. Associations with sepsis, RDS, BPD, PDA, ROP ≥ stage 2, and IVH ≥ grade 2 were not statistically significant. Additional models including complete antenatal corticosteroid course and singleton-only sensitivity analyses yielded materially similar results, with EUGR remaining significantly associated with NEC across all three growth-chart definitions.

**Table 3:** Adjusted association between EUGR (by growth chart) and neonatal morbidities

Comorbidities	Fenton 2013 EUGR aOR (95% CI)	p	IG-21 EUGR aOR (95% CI)	p	Fenton 2025 EUGR aOR (95% CI)	p
Sepsis	3.62 (0.87–15.05)	0.076	2.00 (0.50–8.10)	0.330	1.87 (0.63–5.61)	0.261
RDS	1.67 (0.64–4.34)	0.294	2.51 (0.91–6.95)	0.077	1.54 (0.65–3.62)	0.327
BPD	0.84 (0.33–2.14)	0.709	0.89 (0.35–2.23)	0.796	0.49 (0.20–1.22)	0.125
PDA	1.08 (0.52–2.26)	0.838	0.82 (0.39–1.71)	0.590	0.98 (0.48–1.98)	0.946
ROP	0.71 (0.29–1.73)	0.449	1.25 (0.52–3.02)	0.617	1.13 (0.48–2.70)	0.776
**NEC**	**6.37 (2.07–19.57)**	**0.001**	**5.03 (1.82–13.90)**	**0.002**	**4.96 (1.68–14.64)**	**0.004**
IVH	2.13 (0.61–7.41)	0.236	1.14 (0.34–3.84)	0.836	1.55 (0.47–5.16)	0.476

Data are presented as adjusted odds ratios (aORs) with 95% confidence intervals (CIs) from separate multivariable binary logistic regression models for each morbidity. In each model, EUGR status, as defined by the respective growth chart, was the primary independent variable, adjusted for gestational age at birth (days), birth weight (g), and sex. For severity-based outcomes, dichotomisation was applied as follows: ROP ≥ stage 2, NEC ≥ stage 2, and IVH ≥ grade 2. Two-sided p values are reported; statistically significant *p* values (*p* < 0.05) are shown in bold

*Abbreviations:* *aOR* adjusted odds ratio, *BPD* bronchopulmonary dysplasia, *CI* confidence interval, *EUGR* extrauterine growth restriction, *IG-21* INTERGROWTH-21st, *IVH* intraventricular hemorrhage, *NEC* necrotising enterocolitis, *PDA* patent ductus arteriosus, *RDS* respiratory distress syndrome, *ROP* retinopathy of prematurity

## Discussion

In this single-centre cohort of very preterm infants (24–32 weeks’ gestation), growth chart selection meaningfully altered SGA classification at birth and EUGR classification at discharge. The newly introduced Fenton-2025 curves yielded the highest prevalence of SGA (24.3%) and EUGR (40.8%). The prevalence of SGA was higher with IG-21 (15.8%) than with Fenton-2013 (9.9%), whereas the prevalence of EUGR was higher with Fenton-2013 (33.5%) than with IG-21 (26.1%). Beyond prevalence differences, a consistent nested reclassification structure was observed for both outcomes (Fig. 2).

In prior preterm cohorts, Fenton-2013 generally identified fewer infants as SGA (6.4–15.2%), whereas IG-21 identified more (8.0–13.5%) findings consistent with our results [7, 13, 14]. Compared with IG-21, Fenton-2025 further expanded the number of infants classified as SGA. EUGR rates in prior studies were again similar to our cohort’s classification patterns, with Fenton-2013 reporting higher EUGR (40.2–81.3%) and IG-21 reporting lower EUGR (31.5–72.8%) [5, 6, 11, 12].

Between-curve discrepancies are expected because “standards” and “references” are not interchangeable and arise from different populations and design goals [2–4]. Local, population-specific growth references further contribute to this variability [22, 23]. IG-21 is a prescriptive international standard derived from prospectively collected data under low-risk conditions with standardized anthropometry, aiming to reflect “optimal” growth trajectories [3]. In contrast, Fenton-2013 charts are fetal-based references synthesised from aggregated birth datasets, intended primarily to position size at birth relative to a reference distribution [2]. The Fenton-2025 update further restricts eligibility to reduce distortion from pregnancies linked to abnormal fetal growth, modifying the evidence base and, plausibly, the lower tail of the centile distributions [4]. These methodological refinements in Fenton-2025 likely shifted lower centiles upward, particularly between 24 and 36 weeks’ gestation, which may explain why the updated curves classified additional infants as EUGR, even beyond Fenton-2013, in very preterm infants. Overall, this pattern suggests that Fenton charts are increasingly inclusive, whereas IG-21 remains comparatively selective for preterm infants.

SGA and EUGR classifications, as defined by more inclusionary versus more selective growth charts, are not merely descriptive labels; they may identify different clinical risk groups in very preterm infants [5–7, 11]. Accordingly, the key question is whether the additional infants captured by the Fenton charts are morbidity-enriched or whether they mainly reflect threshold-driven expansion [6]. From this perspective, we compared the clinical characteristics and morbidity profiles of the chart-defined SGA subgroups. In our cohort, the morbidity differences between SGA and non-SGA infants were limited; only sepsis differed consistently across the chart-defined SGA groups, whereas other major morbidities did not.

EUGR at discharge is even more chart- and definition-sensitive than that of SGA. Interpretation of EUGR outcome associations is further complicated by heterogeneity in EUGR definitions across studies (cross-sectional percentile thresholds vs. change in Z-score), which may capture overlapping but non-identical growth phenotypes [5, 6]. Prior comparative studies have reported lower EUGR prevalence with IG-21 and higher prevalence with Fenton-based approaches, with the additional observation that the IG-21-defined group may have more morbidity in some settings [5, 6]. Our nested structure is consistent with this framework: IG-21 identified a more restrictive core group; however, morbidity patterns across chart-defined EUGR groups were broadly comparable. In our cohort, EUGR infants had a more intensive clinical course across all chart definitions, with longer respiratory support, greater TPN exposure, and prolonged hospitalisation. After adjusting for gestational age, birth weight, and sex, EUGR remained consistently associated only with NEC ≥ stage II across all three chart definitions.

Our results have two practical implications. First, growth chart selection should be reported explicitly in both clinical communication and research, as SGA and EUGR are not interchangeable constructs across tools. Second, when comparing cohorts or benchmarking units, prevalence alone is insufficient; it should be complemented by agreement metrics, reclassification patterns, and outcome-linked validation of “incremental” cases. In particular, evaluating whether infants additionally classified by Fenton-2025 (beyond IG-21 and Fenton-2013) show meaningful differences in nutrition-related exposures, complication trajectories, and longer-term growth or neurodevelopment would help determine whether increased case detection improves sensitivity without an undue loss of specificity [2, 6].

This study has several limitations. The retrospective, single-centre design limited causal inference and external generalizability. Discharge-defined EUGR depends partly on discharge timing and local practice and may partly reflect length of stay and clinical stability in addition to growth biology. Dynamic delta Z-score-based definitions were not used. Residual confounding is likely because granular nutritional delivery variables and formal illness severity indices were not uniformly available.

In addition, morbidity analyses were secondary and included multiple outcomes; therefore, these findings should be interpreted as exploratory rather than confirmatory. Multicentre studies with standardised anthropometry, detailed nutritional covariates, and longer-term follow-up are needed to determine whether Fenton-2025 reclassification improves clinically meaningful risk stratification.

## Conclusions

Growth-reference selection in very preterm infants meaningfully alters SGA and EUGR classification, producing non-equivalent case mixes despite generally high agreement. The observed nested structure indicates that IG-21 identifies a restrictive core EUGR group, whereas Fenton-2013 and particularly Fenton-2025 progressively expand ascertainment near the diagnostic thresholds. In our adjusted analyses, NEC remained the most consistent morbidity associated with EUGR across chart definitions. These findings support transparent, chart-specific reporting and emphasise the need for outcome-linked validation of reclassified infants to determine whether more inclusionary definitions provide clinically meaningful risk stratification or primarily broaden classification without clear downstream benefit.

## Supplementary Information

Additional file 1.

## Data Availability

The datasets generated and/or analysed during the current study are not publicly available due to institutional data protection policies but are available from the corresponding author on reasonable request.

## Abbreviations

AGA: Appropriate for gestational age
aOR: Adjusted odds ratio
BPD: Bronchopulmonary dysplasia
CI: Confidence interval
EUGR: Extrauterine growth restriction
F13: Fenton-2013
F25: Fenton-2025
IG-21: INTERGROWTH-21st Project (International Fetal and Newborn Growth Consortium for the 21st Century)
IQR: Interquartile range
IUGR: Intrauterine growth restriction
IVH: Intraventricular haemorrhage
LGA: Large for gestational age
MV: Mechanical ventilation
NEC: Necrotising enterocolitis
NICU: Neonatal intensive care unit
PDA: Patent ductus arteriosus
Po: Observed agreement
PPROM: Preterm premature rupture of membranes
RDS: Respiratory distress syndrome
ROP: Retinopathy of prematurity
SGA: Small for gestational age
TPN: Total parenteral nutrition
