# Supplementary material for: Reclassification of SGA and EUGR in very preterm infants after the introduction of Fenton-2025: a retrospective cohort comparison with INTERGROWTH-21st and Fenton-2013
Source: BMC Pediatr. 2026 Jun 25;26:855. doi: 10.1186/s12887-026-07215-5 (PMC13560381; doi:10.1186/s12887-026-07215-5)
Supplement: Supplementary file 1 [file 12887_2026_7215_MOESM1_ESM.docx]

**Additional file 1.** Pairwise Agreement Between Growth Charts for SGA/AGA/LGA and EUGR Classification

| **Comparison** | **Po** | **PABAK** | **κ** | **95% CI (κ)*** | **Agreement (Landis–Koch)** |
| --- | --- | --- | --- | --- | --- |
| SGA/AGA/LGA | | | | | |
| F13 vs IG21 | 0.915 | 0.873 | 0.759 | 0.656–0.847 | Substantial |
| IG21 vs F25 | 0.890 | 0.835 | 0.757 | 0.671–0.835 | Substantial |
| F13 vs F25 | 0.835 | 0.753 | 0.616 | 0.517–0.709 | Substantial |
| EUGR | | | | | |
| F13 vs IG21 | 0.926 | 0.853 | 0.825 | 0.749–0.894 | Almost perfect |
| IG21 vs F25 | 0.853 | 0.706 | 0.678 | 0.588–0.764 | Substantial |
| F13 vs F25 | 0.926 | 0.853 | 0.843 | 0.775–0.905 | Almost perfect |

*Abbreviations:* CI, confidence interval; F13, Fenton-2013; F25, Fenton-2025; IG21, INTERGROWTH-21st; κ, Cohen’s kappa coefficient; PABAK, prevalence-adjusted bias-adjusted kappa; Po, observed agreement.

Footnote: Po denotes the observed agreement (proportion of identical classifications). PABAK was calculated as (k × Po − 1) / (k − 1), where k denotes the number of classification categories. Cohen’s κ was used to quantify pairwise agreement between growth charts. Ninety-five percent confidence intervals for κ were obtained using percentile bootstrap with 10,000 resamples. Agreement strength was interpreted according to the Landis–Koch benchmarks.
